# Sex differences in patients with COVID-19 after bariatric surgery: a multicenter cross-sectional study

**DOI:** 10.3389/fpubh.2023.1293318

**Published:** 2024-01-15

**Authors:** Senlin Wang, Qiubai Jang, Han Wang, Yunning Yang, Min Ruan, Juan Yu, Xiuying Li, Dan Luo

**Affiliations:** ^1^Center for Obesity and Metabolic Health, Department of General Surgery, The Third People’s Hospital of Chengdu, The Affiliated Hospital of Southwest Jiaotong University, Chengdu, China; ^2^Department of General Surgery, Center for Obesity and Metabolic Health, The Third People’s Hospital of Chengdu, The Affiliated Hospital of Southwest Jiaotong University, Chengdu, China; ^3^Mianyang Central Hospital, Mianyang, China; ^4^First Affiliated Hospital of Air Force Military Medical University, Xi’an, China; ^5^Dazhou Central Hospital, Dazhou, China

**Keywords:** obesity, bariatric surgery, sex, COVID-19, cross-sectional study

## Abstract

**Objectives:**

This multicenter, cross-sectional study aimed to investigate whether sex differences persist among patients who have undergone bariatric surgery and tested positive for the coronavirus disease (COVID-19).

**Methods:**

We conducted a multicenter cross-sectional study via an online electronic questionnaire to collect data. Categorical data were presented as absolute and relative frequencies. Data for continuous variables were expressed as mean and standard deviation (SD) or median [interquartile range (IQR)]. We employed ordered logistic regression to assess whether females had higher odds of an increased self-reported duration of the most severe symptom compared to males. Using a modified Poisson regression model with robust standard errors to assess the differences in clinical characteristics among COVID-19 cases.

**Results:**

Statistical analysis revealed significant differences in the prevalence rates of various comorbidities. Among participants who reported their temperature during COVID-19 infection, more than half engaged in vitamin supplementation and regular exercise, while 4.2% remained asymptomatic. The probability of females experiencing a longer duration of severe symptoms increased compared to males [adjusted Odds Ratio (aOR) = 1.92, 95% confidence interval (CI) 1.73–2.12]. In the multivariate mixed-effects Poisson regression analysis, compared to males, females exhibited a lower prevalence rate of asymptomatic infection [adjusted prevalence ratio (aPR 0.40, 95% CI 0.28–0.58), lower prevalence of infection without therapeutic medication use (aPR 0.76, 95% CI 0.70–0.82), and lower prevalence of multiple infections (aPR 0.39, 95% CI 0.20–0.74)].

**Conclusion:**

This cross-sectional study indicates the persistence of sex differences among patients with COVID-19 who have undergone bariatric surgery. Further research is needed to explore the underlying factors contributing to this disparity.

## 1 Introduction

As of November 2023, the novel coronavirus disease (COVID-19) has resulted in over 700 million confirmed cases and approximately 7 million deaths worldwide (1). Studies have shown that men are more likely to experience severe symptoms and a higher mortality rate from COVID-19 than women (2–4). To address this issue, the World Health Organization (WHO) has recommended collecting data disaggregated by sex in the COVID-19 Strategic Preparedness and Response Plan (SPRP) (5).

The relationship between a history of bariatric surgery, sex differences, and severe outcomes in COVID-19-positive patients is still under investigation. Currently, many clinical and epidemiological studies have demonstrated disproportionate sex-based discrepancy in the severity of COVID-19 symptoms, with men twice as likely to develop severe symptoms or die from the virus compared to women (6, 7). The cumulative effects of biological factors, lifestyle factors, social structural factors, etc., render males a high-risk population (4, 8, 9). However, one study reported no statistically significant differences in symptom presence, hospitalization requirement, intensive care unit (ICU) admission, and invasive ventilation among patients infected with COVID-19 before and after sleeve gastrectomy (10). Another study conducted in western Sweden reported that the risk of ICU admission was associated with a higher body mass index (BMI) only among women (11). Interestingly, studies have also shown that patients with a history of bariatric surgery have lower risks of emergency admission, mechanical ventilation, prolonged ICU stay, and mortality in the context of COVID-19 infection (12–14).

However, sex-specific studies in Chinese post-bariatric patients are still limited. Our study aimed to explore whether sex differences persist among patients with COVID-19 after bariatric surgery by conducting a questionnaire survey based on the basic characteristics, comorbidities, infection, and living habits.

## 2 Methods

### 2.1 Study population and design

This is a multicenter, cross-sectional study involving eight medical centers, conducted from December 1, 2022, to January 30, 2023. Each patient undergoing bariatric surgery treatment was identified by a nursing staff member at each site using the hospital’s patient management system. They were invited via telephone to participate in an online electronic questionnaire. The collected data were stored in an online database that was protected and accessible only to authorized personnel. The data supporting this study’s findings are available from the corresponding author upon reasonable request.

The survey included three main parts: (1) basic information about patients, including age, sex, preoperative BMI and postoperative BMI, and date of surgery; (2) COVID-19-related information, including COVID-19 vaccination status, symptoms after infection, drug use, time to negative conversion, etc.; (3) changes in body weight, physical exercise habits, and nutrient supplementation during infection. The questions about the questionnaire are detailed in Supplementary Table S1. Inclusion Criteria: (1) The individuals included in the study are those who have undergone weight loss surgery and subsequently confirmed to be infected with COVID-19 through laboratory testing, either by real-time (Polymerase Chain Reaction) PCR fluorescence method or Antigen Detection; (2) Due to a significant increase in the number of infections in a short period after the Chinese government lifted the lockdown measures for COVID-19 after December 1, 2022, we only analyzed individuals infected after December 1, 2022, to minimize recall bias. Exclusion Criteria: (1) Participants who did not provide informed consent to participate in the study; (2) Individuals who incompletely filled out the questionnaire.

**Figure 1:**
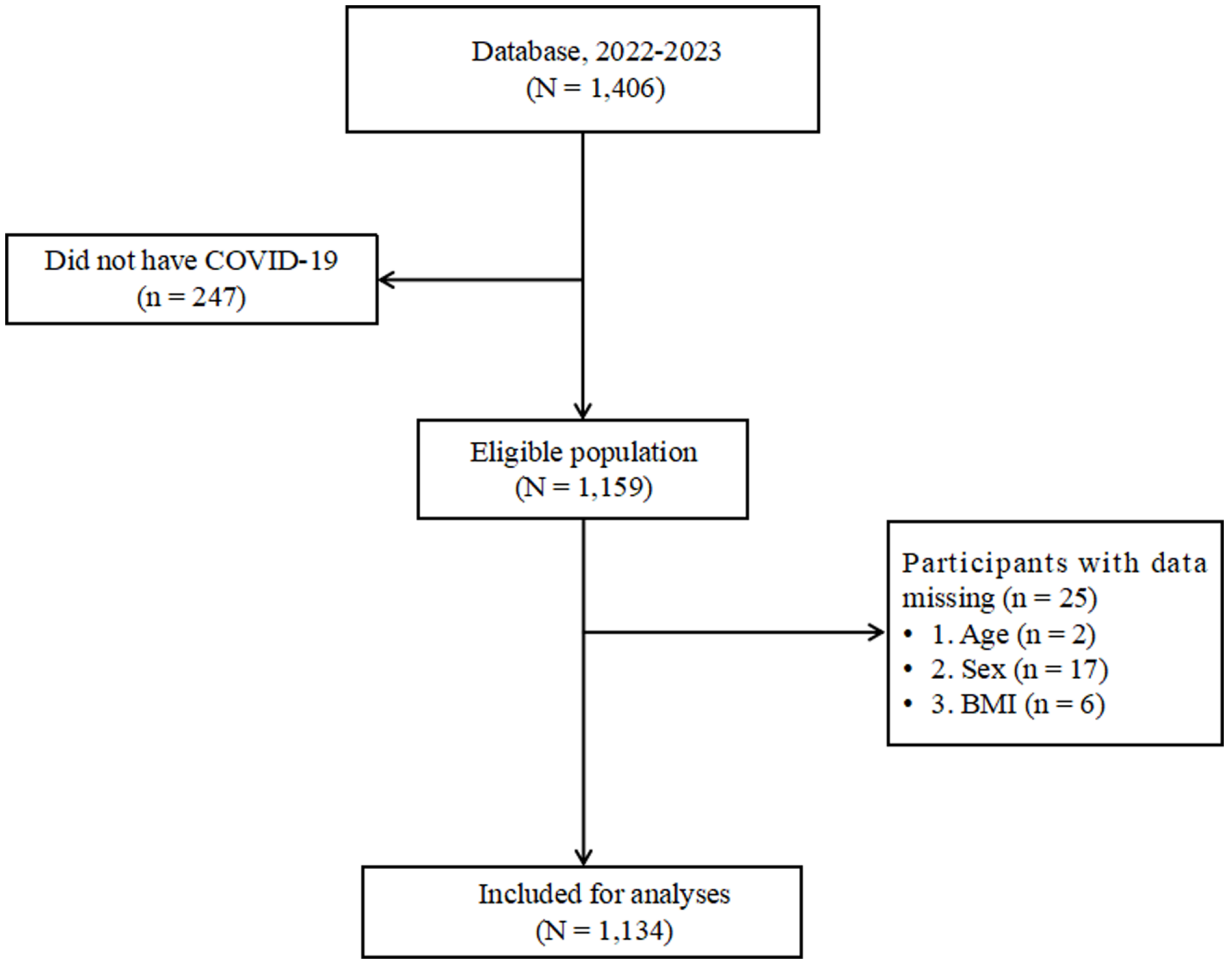
Flowchart of eligibility of study participants in the Database. BMI, body mass index; Database, Database of Weight Loss and Metabolism Center.

The disease characteristics of COVID-19 in these patients were evaluated. Out of the 1,710 questionnaires distributed, 1,406 survey reports were collected, resulting in a response rate of 82.22%. After excluding questionnaires with missing data on COVID-19 and outliers (Figure 1), the final analysis included 1,134 participants, representing 66.32% of the total initial sample.

### 2.2 Definition of outcomes, exposures, and covariates

This study focuses on several outcomes of interest. The duration of self-reported severe symptoms, considered the primary endpoint of our investigation, was examined. Specifically, following the pre-designed questionnaire, participants were asked the following specific question during the survey: “How long did the most severe symptoms you experienced during the infection persist? Please choose: A. Less than 24 h, B. One day, C. Two days, D. Three days, E. More than 3 days.” Secondary outcomes include investigating vaccination status, participation in physical activities during the infection, vitamin C supplementation, composite vitamin supplementation, protein powder supplementation, asymptomatic infection status, use of COVID-19-related medications during the infection, and the total number of reported COVID-19 infections by participants at the time of data submission. The exposure variable is sex, with males serving as the reference group in all regression analyses.

In our study, the primary focus revolved around the severity of participants’ infection with COVID-19. Therefore, relevant variables were identified based on professional knowledge (15, 16), and their associations with the self-reported duration of severe symptoms of COVID-19 were examined as the main outcome. Employing a historical confounding approach, a multivariate regression model incorporated age (years) (17), BMI group (18), education level (19), marital status (20), smoking status (21), alcohol consumption (22), bariatric surgery type (SG, RYGB, and others) (23), hypertension (24), and diabetes (25). Due to the skewed distribution of BMI data, we grouped the values into intervals with uniform spacing of 5, namely (<20, 20–25, 25–30, 30–35, 35–40, 45–50, 50–55, and 55–60 kg/m^2^). It should be emphasized that we combined the <20 category with the BMI 20–25 category, creating a <25 BMI category, which was used as our reference category. Due to limited sample size in certain categories, making it challenging to achieve sufficient fit in regression modeling, the education level was grouped into four categories: high school and below (including high school, junior high, and elementary), college, undergraduate, and graduate degree. Similarly, surgical procedures were categorized into four groups based on current mainstream techniques: SG, RYGB, and others (SG + JJB, OAGB, BIDs, and unknown) (26).

### 2.3 Statistical analysis

Categorical data were presented as absolute and relative frequencies. Continuous variables were presented as mean and standard deviation (SD). Due to the non-normal distribution of BMI, percentage of excess weight loss (EWL%), percentage of total weight loss (TWL%), and the time to negative variables (the time participants self-reported from the onset of self-discovered COVID-19 infection to a negative result in COVID-19 real-time PCR fluorescence method or Antigen Detection), these variables are expressed as median [interquartile range (IQR)]. For BMI, EWL%, TWL% and the time to negative variables, the Wilcoxon rank-sum test (ranksum test) was employed for independent sample comparison. The TWL% and EWL% can be utilized to the indirectly approximate effectiveness of bariatric surgery, offering a quantitative measure of the extent of individual weight reduction. Continuous variables were analyzed using Student’s *t*-test with sex as the grouping variable. For categorical variables such as education level, marital status, alcohol consumption, smoking status, and comorbidities (excluding coronary heart disease, hypothyroidism, and osteoarthritis), Pearson’s chi-square test was employed. Additionally, for categorical variables such as coronary heart disease, hypothyroidism, and osteoarthritis, Fisher’s exact test was utilized to compare baseline characteristics and infection status, given that theoretical frequencies were less than 5 in some categories.

In the analysis of the primary outcome, we employed ordered logistic regression to assess whether females had higher odds of an increased self-reported duration of the most severe symptom compared to males. The Parallel Lines Assumption was assessed using the Brant test, where *p* > 0.05 was considered indicative of the suitability for adopting the ordered logistic regression (27). Under the assumption of parallel lines, and recognizing the unique characteristics of patients from each center, which render them not entirely independent, we utilized a mixed-effects model to adjust for the impact of center-specific effects. Therefore, for our primary outcome, we applied a mixed-effects ordered regression model. The effect size in the univariate mixed-effects ordered regression model was expressed as Odds Ratio (OR), and 95% confidence intervals (CIs). In the multivariate mixed-effects ordered regression model, the effect size was represented as Adjusted OR (aOR), and 95% CIs. In Model 1, the association between gender and self-reported duration of severe symptoms was assessed without controlling for any variables. In Model 2, age (years), BMI group were added based on Model 1. Model 3 further included education level, marital status, smoking status, and alcohol consumption, bariatric surgery type, hypertension, and diabetes.

The secondary outcomes were exploratory. Following univariate analysis, variables showing differences in clinical characteristics between males and females after infection were explored. These variables included self-reported asymptomatic infection, self-reported non-usage of therapeutic drugs at the time of infection, and the frequency of COVID-19 infections. In accordance with our research design, utilizing the Prevalence ratio (PR) for analysis is more intuitive, as it allows for a direct comparison of raw morbidity rates (28). Since patients from each center possess unique characteristics, rendering them not entirely independent, we employed a mixed-effects model to adjust for the impact of center-specific effects. Ultimately, our regression analysis utilized a modified Poisson regression model with robust standard errors to evaluate the effect size of infection status between males and females. In the multivariate mixed-effects Poisson regression model, the effect size was denoted as Adjusted PR (aPR) with associated 95% CIs. Adjusted for age (years), BMI group, education level, marital status, smoking status, alcohol consumption, bariatric surgery type, hypertension, and diabetes.

All statistical tests were two-tailed and *p* < 0.05 was considered statistically significant. All statistical analyses were performed using STATA 17.0 (Stata Corp LLC, College Station, Texas, US).

## 3 Results

### 3.1 Baseline characteristics of the participants

Of 1,134 participants, the mean age was 33.64 (SD, 8.47) years including 852 females (75.1%) and 282 males (24.9%) (Table 1). The overall BMI of the participants ranged from 17.99 to 58.95 kg/m^2^, with a median of 26.04 (IQR: 23.62–29.41). Survey found that among the participants, 61.2% were married, 37.7% had a bachelor’s degree, 67.9% had never consumed alcohol, and 71.4% had never smoked. The median interval between the completion of surgery and the most recent occurrence of COVID-19 is 9 months (IQR: 4–16). Among the participants, the highest incidence was observed for hypertension, reaching 45.4%. This was followed by hyperlipemia and sleep apnea syndrome, with incidences of 38.7%. Most commonly performed bariatric surgical technique was sleeve gastrectomy (SG) (79.6%). The majority of participants included in the study were from The Third People’s Hospital of Chengdu (*n* = 668), with only 14 individuals from the First Affiliated Hospital of Air Force Military Medical University and Mianyang Central Hospital, as illustrated in Supplementary Figure S1.

**Table 1:** Baseline characteristics of patients infected with COVID-19 after bariatric surgery.

Characteristics	Male	Female	Total	* *Value of p*
	(*N* = 282)	(*N* = 852)	(*N* = 1,134)	
**Age, Mean (SD), years**	33.77 (8.63)	33.59 (8.42)	33.64 (8.47)	0.761
**BMI, Median (IQR), kg/m** ^**2**^	27.58 (24.52–30.67)	25.66 (23.32–28.92)	26.04 (23.62–29.41)	<0.001
**TWL, Median (IQR), %**	27.78 (21.75–33.57)	26.67 (21.05–32.14)	26.89 (21.18–32.33)	0.063
**EWL, Median (IQR), %**	80.61 (64.60–104.10)	93.04 (68.70–119.44)	89.77 (68.11–116.14)	<0.001
**Education level**				0.001
High school and below	77 (27.3%)	273 (32.0%)	350 (30.9%)	
Junior college	73 (25.9%)	246 (28.9%)	319 (28.1%)	
Undergraduate	113 (40.1%)	314 (36.9%)	427 (37.7%)	
Postgraduate	19 (6.7%)	19 (2.2%)	38 (3.4%)	
**Marital status**				0.027
Divorce	26 (9.2%)	71 (8.3%)	97 (8.6%)	
Married	154 (54.6%)	540 (63.4%)	694 (61.2%)	
Unmarried	102 (36.2%)	241 (28.3%)	343 (30.2%)	
**Alcohol consumption**				<0.001
Current	37 (13.1%)	27 (3.2%)	64 (5.6%)	
Former	77 (27.3%)	183 (21.5%)	260 (22.9%)	
Never	168 (59.6%)	642 (75.4%)	810 (71.4%)	
**Smoking status**				<0.001
Current	104 (36.9%)	86 (10.1%)	190 (16.8%)	
Former	63 (22.3%)	111 (13.0%)	174 (15.3%)	
Never	115 (40.8%)	655 (76.9%)	770 (67.9%)	
**Procedure**				<0.001
SG	204 (72.3%)	699 (82.0%)	903 (79.6%)	
SG + JJB	49 (17.4%)	113 (13.3%)	162 (14.3%)	
RYGB	19 (6.7%)	22 (2.6%)	41 (3.6%)	
OAGB	3 (1.1%)	0 (0.0%)	3 (0.3%)	
BIDs	0 (0.0%)	1 (0.1%)	1 (0.1%)	
Unknown	7 (2.5%)	17 (2.0%)	24 (2.1%)	
**Interval time, Median (IQR), months**	9 (4–16)	8 (4–16)	8 (4–16)	0.925
**Comorbidities**				
Hypertension	128 (45.4%)	148 (17.4%)	276 (24.3%)	<0.001
Coronary heart disease	9 (3.2%)	4 (0.5%)	13 (1.1%)	<0.001
Hyperlipemia	109 (38.7%)	191 (22.4%)	300 (26.5%)	<0.001
Gout	81 (28.7%)	29 (3.4%)	110 (9.7%)	<0.001
Diabetes	77 (27.3%)	162 (19.0%)	239 (21.1%)	0.003
NAFLD	85 (30.1%)	216 (25.4%)	301 (26.5%)	0.114
Sleep apnea syndrome	109 (38.7%)	317 (37.2%)	426 (37.6%)	0.664
Tristimania	11 (3.9%)	55 (6.5%)	66 (5.8%)	0.112
Polycystic ovarian syndrome	0(0.0%)	272 (31.9%)	272 (23.9%)	<0.001
Nephrotic syndrome	7 (2.5%)	6 (0.7%)	13 (1.1%)	0.015
Hypothyroidism	5 (1.8%)	55 (6.5%)	60 (5.3%)	0.002
Osteoarthritis	1 (0.4%)	21 (2.5%)	22 (1.9%)	0.026
Acanthosis nigricans	49 (17.4%)	112 (13.1%)	161 (14.2%)	0.078

COVID-19, coronavirus disease; %EWL, percentage of excess weight loss; %TWL, percentage of total weight loss; NAFLD, Non-alcoholic fatty liver disease. Procedure: Biliopancreatic Diversion with Duodenal Switch (BIDs), One-anastomosis gastric bypass (OAGB), Roux-en-Y Gastric Bypass (RYGB), Sleeve Gastrectomy (SG), sleeve gastrectomy with jejunal-jejunal bypass (SG + JJB). Interval time: the time from the end of surgery to the completion of the questionnaire was recorded. ^*^p value by Student’s *t*-test for continuous variables, chi-square test for categorical variables, and Fisher exact probability method test for categorical variables when the theoretical frequency in the count data is less than or equal to 5.

Single-factor analysis revealed that, compared to male participants, female participants had lower BMI values, higher EWL% indices, were more likely to be married, and had no history of alcohol consumption and smoking. In the analysis of comorbidities, female participants showed a relatively higher incidence of Hypothyroidism and Osteoarthritis compared to male participants. However, the incidence rates of Hypertension, Coronary heart disease, Hyperlipemia, Gout, Diabetes, and Nephrotic syndrome were lower in female participants than in male participants (all *p* < 0.001).

### 3.2 Effects of COVID-19 on the bariatric surgery population

#### 3.2.1 A comparison of outcomes in male and female COVID-19 cases

**Figure 2:**
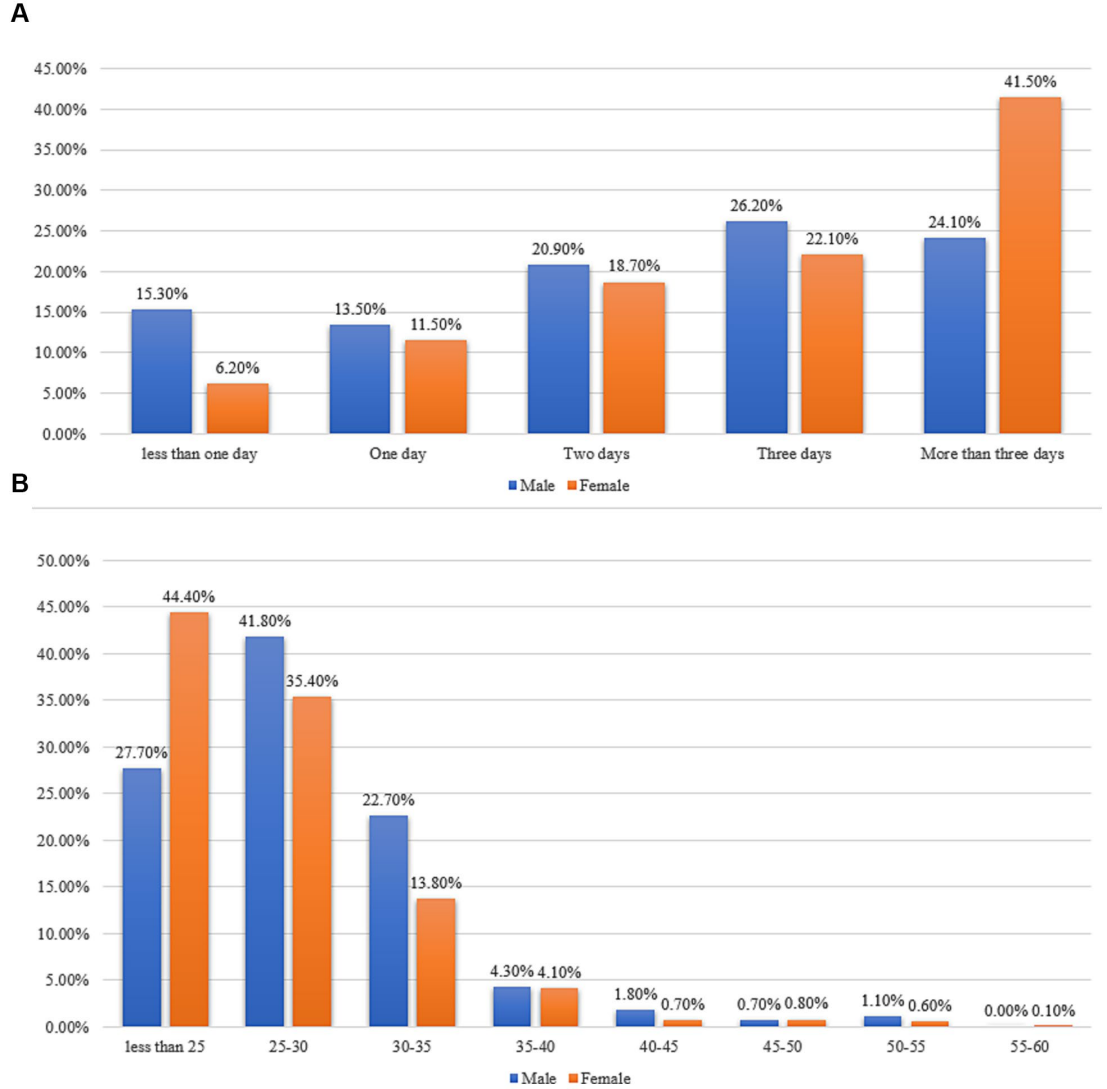
Relative frequency bar charts. **(A)** Sex-specific relative frequency bar chart for self-reported symptom duration. **(B)** Sex-specific relative frequency bar chart for different BMI groups.

In 1,134 patients with COVID-19, only 806 (71.1%) reported their body temperature, including 199 males and 607 females (Table 2). 98.6% of respondents reported having been infected with the virus, 1.2% reported being infected twice, and 0.2% reported being infected three times. Over half of the participants reported taking vitamin C, multivitamins, or engaging in physical exercise during infection (58.2, 54.1, and 57.7%, respectively). However, only 22.5% of respondents consistently took protein powder during the pandemic. In our surveyed population, 3.4% of respondents stated that they had never received any brand of COVID-19 vaccine. Interestingly, 4.2% of the infected individuals were asymptomatic, and 16.9% did not take any COVID-19 medications. The reported symptom intensity was based on their self-assessment. The overall median days to negative were 20 (IQR: 16–22) days, and there was no significant difference observed in the median days to negative between the two groups. Figure 2 displays a bar chart of relative frequencies grouped by sex. It can be observed that females have the highest prevalence rates of severe symptom duration exceeding 3 days, at 41.5%. In contrast, males report the highest prevalence rates for a duration of 3 days, reaching 24.1%. Female participants exhibit the highest prevalence rates in the BMI <25 kg/m^2^ range, at 44.40%, while males have the highest prevalence rates in the BMI (25–30 kg/m^2^) range, at 41.8%.

**Table 2:** A comparison of outcomes in male and female COVID-19 cases on the bariatric surgery population in China.

Characteristics	Male	Female	* *Value of p*
**Temperature, Mean (SD), °C**	38.91 (0.83)	38.99 (0.78)	0.220
**Days to negative, Median (IQR)**	20 (16–22)	20 (16–22)	0.749**
**Have been vaccinated against COVID-19, yes**	271 (96.1%)	825 (96.8%)	0.447
**Exercise, yes**	173 (61.3%)	481 (56.5%)	0.293
**Vitamin C supplementation, yes**	170 (60.3%)	490 (57.5%)	0.413
**Taking multivitamins during infection, yes**	159 (56.4%)	455 (53.4%)	0.384
**Taking protein powder during the infection, yes**	67 (23.8%)	188 (22.1%)	0.555
**No symptoms, yes**	21 (7.4%)	27 (3.2%)	0.002
**No medication was taken, yes**	61 (21.6%)	131 (15.4%)	0.015
**Duration of severe symptoms**			<0.001
≤one day	43 (15.3%)	53 (6.2%)	
One day	38 (13.5%)	98 (11.5%)	
Two days	59 (20.9%)	159 (18.7%)	
Three days	74 (26.2%)	188 (22.1%)	
More than 3 days	68 (24.1%)	354 (41.5%)	
**Number of infections with COVID-19**			0.003
Once	273 (96.8%)	845 (99.2%)	
Two or three times	9 (3.2%)	7 (0.8%)	

SD, standard deviation; CI, confidence interval; PR, Prevalence ratio. No medication was taken: did not take any medication to treat the disease caused by COVID-19. Temperature: collected only 799 valid responses, of which the male-to-female ratio was (197:602). Days to negative: collected only 759 valid responses, of which the male-to-female ratio was (189:570). ^*^P value by ANOVA for continuous variables, chi-square test for categorical variables. **p-value of categorical/numerical variables calculated with the *U* test (Mann–Whitney).

#### 3.2.2 Sex differences in the duration of severe symptoms after self-reported infection: a mixed-effects ordered logistic regression analysis

Table 3 reports the association between gender and the duration of self-reported severe symptoms. Without controlling for any covariates, the probability of females experiencing a longer duration of severe symptoms increased compared to males (OR = 1.99, 95% CI 1.84–2.15, Model 1). Even after controlling for age and BMI, this relationship persisted (aOR = 2.02, 95% CI 1.86–2.18, Model 2). After controlling for age (years), BMI group, education level, marital status, smoking status, alcohol consumption, bariatric surgery type, hypertension, and diabetes, this finding remained robust (aOR = 1.92, 95% CI 1.73–2.12, Model 3).

**Table 3:** Mixed-effects ordered logistic regression analysis of duration of severe symptoms among COVID-19 cases in different sex in China.

Primary outcome	Model 1		Model 2		Model 3	
	**OR (95% CI)**	***P*-values**	**aOR (95% CI)**	***P*-values**	**aOR (95% CI)**	***P*-values**
**Sex (ref: male)**	1.99 (1.84–2.15)	<0.001	2.02 (1.86–2.18)	<0.001	1.92 (1.73–2.12)	<0.001
**Age**			1.00 (0.99 to 1.00)	0.851	0.99 (0.99–1.00)	0.024
**BMI group (ref: <25 kg/m** ^**2**^ **)**						
**25–30**			1.03 (0.90–1.19)	0.636	1.04 (0.89–1.21)	0.612
**30–35**			1.08 (0.88–1.35)	0.488	1.10 (0.88–1.37)	0.397
**35–40**			0.73 (0.53–1.02)	0.062	0.74 (0.53–1.03)	0.074
**40–45**			0.95 (0.28–3.17)	0.931	1.02 (0.36–2.94)	0.963
**45–50**			1.21 (0.31–4.09)	0.780	1.15 (0.26–5.04)	0.850
**50–55**			1.75 (0.75–4.07)	0.190	1.77 (0.94–3.33)	0.075
**55–60**			0.90 (0.83–0.97)	0.006	0.94 (0.77–1.13)	0.493
**Education level (ref: high school and below)**						
Junior college					1.01 (0.81–1.25)	0.947
Undergraduate					1.11 (0.92–1.35)	0.276
Postgraduate					0.99 (0.42–2.35)	0.978
**Marital Status (ref: divorce)**						
Married					1.01 (0.86–1.18)	0.937
Unmarried					0.88 (0.72–1.07)	0.200
**Alcohol consumption (ref: Current)**						
Former					0.80 (0.65–0.97)	0.015
Never					0.80 (0.62–1.02)	0.073
**Smoking Status (ref: Current)**						
Former					1.51 (1.17–1.97)	0.002
Never					1.61 (1.38–1.88)	<0.001
**Surgery type (ref: SG)**						
RYGB					1.17 (0.82–1.65)	0.396
Others					0.94 (0.75–1.17)	0.580
**Hypertension (ref: no)**					1.20 (1.00–1.45)	0.054
**Diabetes (ref: no)**					1.05 (0.74–1.48)	0.778
^§^ **Variance of the group variable**	<0.001		<0.001		<0.001	

OR, Odds ratio; CI, confidence interval. Model 1: unadjusted; Model 2: adjusted for age (years), BMI group; Model 3: adjusted for variables in Model 2 plus education level, marital status, smoking status, alcohol consumption, bariatric surgery type, hypertension, and diabetes. The OR is calculated through mixed-effects ordered logistic regression, with sex as the exposure variable, using (male) as the reference. ^§^Variance of the group variable indicates the magnitude of variability among individual centers within the groups.

#### 3.2.3 Secondary outcomes of mixed-effects Poisson regression analysis on clinical characteristics of COVID-19 cases

Table 4 presents the results of multivariate mixed-effects Poisson regression analysis for secondary outcomes. The study revealed that, compared to males, females had a lower aPR for asymptomatic infection (aPR 0.40, 95% CI 0.28–0.58), lower prevalence of infection without therapeutic medication use (aPR 0.76, 95% CI 0.70–0.82), and lower prevalence of multiple infections (aPR 0.39, 95% CI 0.20–0.74). These findings remained robust after controlling for covariates (Supplementary Table S2).

**Table 4:** Mixed-effects Poisson regression analysis of clinical characteristics among COVID-19 cases in different sex in China.

Secondary outcomes	aPR (95% CI) ^#^	*P-*values
**No symptoms, yes**		
Males (ref) vs. Females	0.40 (0.28–0.58)	<0.001
**No medication was taken, yes**		
Males (ref) vs. Females	0.76 (0.70–0.82)	<0.001
**Number of infections with COVID-19, (two/three times)**		
Males (ref) vs. Females	0.39 (0.20–0.74)	0.004

PR, Prevalence ratio; CI, confidence interval. #: adjusted for age (years), BMI group, education level, marital status, smoking status, alcohol consumption, bariatric surgery type, hypertension, and diabetes. *P-values obtained with Generalized Linear Models (GLM), Poisson family, log-link function, robust variance. The aPR is calculated through Multivariate-factor mixed-effects Poisson regression, with sex as the exposure variable, using (male) as the reference.

#### 3.2.4 Symptoms of COVID-19 infection

**Figure 3:**
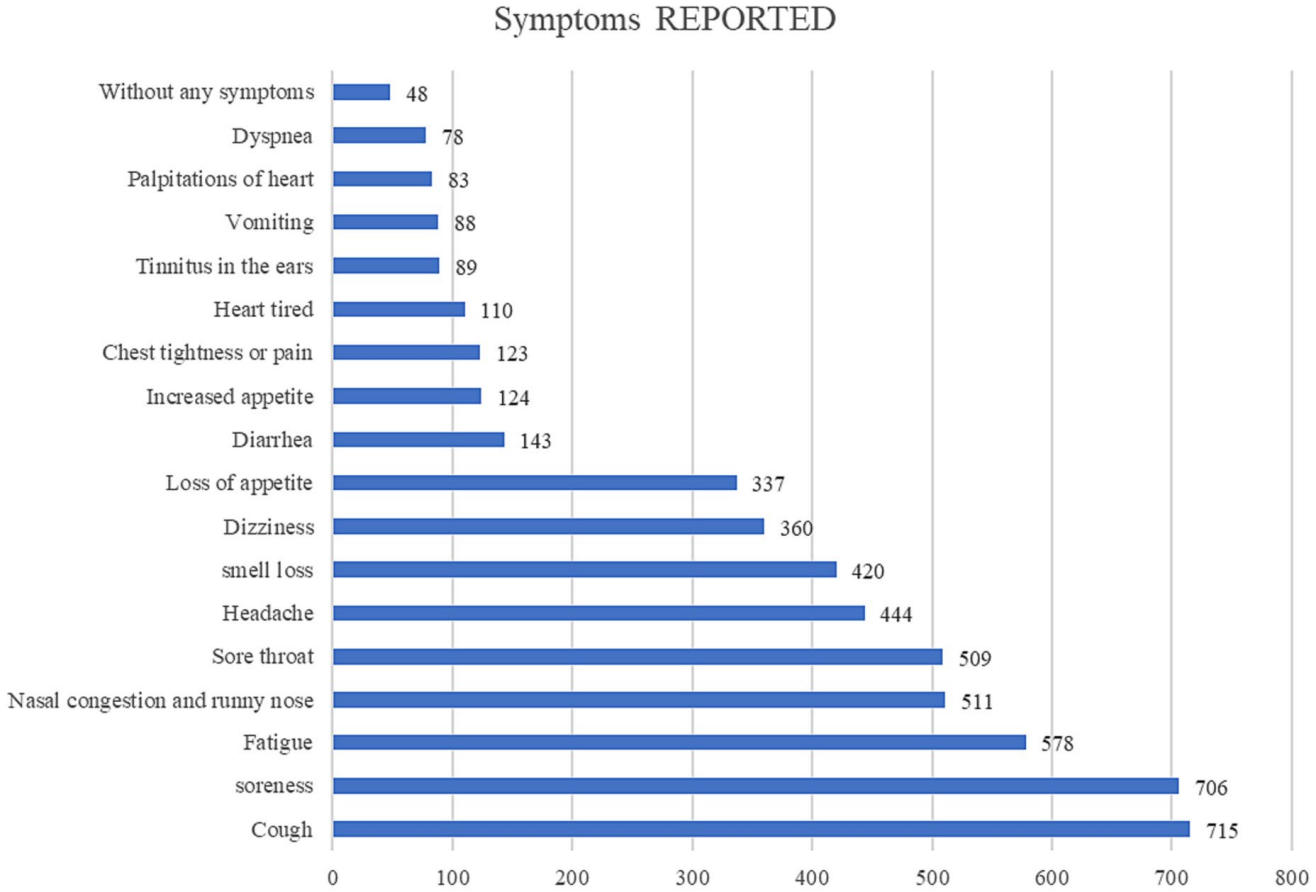
Report results of symptoms of COVID-19 infection after bariatric surgery.

According to Figure 3, we can find that soreness, cough, and fatigue are the most common symptoms of infection reported. Of the reports received on the characteristics of symptoms following COVID-19 infection, 4.2% reported not having any symptoms while infected (Supplementary Table S3). Diarrhea, fatigue, chest tightness or pain, dyspnea, palpitation, and tinnitus had no significant difference between men and women in univariate analysis, while cough, sore throat, vomiting, soreness, loss of taste and smell, nasal congestion and runny nose, dizziness, headache, heart fatigue, increased appetite, and decreased appetite had significant difference between men and women in univariate analysis.

## 4 Discussion

In this survey, we investigated Chinese bariatric surgery patients with COVID-19 and identified persistent sex differences, emphasizing a greater risk of prolonged severe symptoms in females. Compared to males, females exhibited a lower prevalence of asymptomatic infections, a lower prevalence of infection without therapeutic medication use, and a lower prevalence of multiple infections.

From the perspective of strict weight loss, our data showed that %EWL and %TWL reached the expected targets, suggesting that the surgical procedure was successful (29, 30). In this context, it is reasonable to explore the impact of bariatric surgery on people with COVID-19 (31). The relationship between social factors (education level, marital status) and COVID-19 outcomes has been previously studied. Low education level (32, 33) and being single (34) is associated with severe COVID-19 severity. This feature is reflected in our report. Of note, whether lifestyle factors influence the severity of COVID-19 is controversial. One study in China reported prevalence rates of smoking, alcohol consumption, and Betel nut chewing of 26.4, 7.1, and 19.0%, respectively, with no significant association between these lifestyle factors and the severity of COVID-19 (35, 36).

However, some studies support the impact of lifestyle-related factors on individuals with COVID-19. A community-based cohort study of 387,109 adults in the United Kingdom showed that simple lifestyle changes can reduce the risk of serious infection (37). Another study suggested that genetic liability to smoking may contribute to an increased risk for a severe course of COVID-19 (38). Our findings found sex differences in the prevalence of smoking, and alcohol consumption reported by patients. However, we believe this difference in lifestyle may strengthen the effect of lifestyle factors after discussion by sex (4, 39, 40). There was also a significant sex shift in comorbidities in the population we investigated, with a greater prevalence of comorbidities in males than in females. Several studies have proved that comorbidities affect the prognosis of COVID-19 (41–43). From this point of view, in the population after weight loss, more attention should be paid to men.

During COVID-19, there was no difference between men and women in adherence to exercise or regular nutritional supplements. Generally, the proportion of vitamin C and multivitamin supplementation was higher than that of protein powder. Several studies in populations with previous bariatric surgery have reported adherence rates ranging from 60 to 92% (44–46). Our reported adherence was below this level, indicating that COVID-19 hurt adherence to nutritional supplements in this population. Notably, 7.4% of men reported no symptoms during infection, compared to a smaller proportion of women (3.2%). Our results are not consistent with published studies. A recent meta-analysis showed that children and women are more likely to present asymptomatic cases of COVID-19 and may be unknown carriers of SARS-CoV-2 (47). Interestingly, 16.9% reported not taking any medication during infection, with a male bias indicating that some symptomatic infected people did not take any medicine to treat Severe Acute Respiratory Syndrome Coronavirus 2 (SARS-CoV-2) infection. As there are no excellent comparative cases, the adoption of this result requires caution. However, we emphasize that we should not reduce our attention to asymptomatic infection in men after bariatric surgery (48).

Moreover, compared to males, females have a higher risk of experiencing prolonged severe symptoms. Although this cannot be directly equated to assessing the severity of the infection, it indirectly confirms the vulnerability of females. Our study findings align with the research conducted by Francesca Fortunato and colleagues (49). Studies have shown that women’s immune system differs from men’s, which will produce a stronger immune response, leading to virus clearance (50). But from the perspective of long-term symptoms, women are more likely to occur than men (51, 52). In our report, women had a higher incidence of typical symptoms (cough, sore throat, nasal congestion, runny nose, etc.) than men, but there was no difference in severe symptoms (dyspnea) between men and women. In contrast, results from the International Severe Acute Respiratory and Emerging Infection Consortium (ISARIC) prospective multinational observational study showed that women were less likely to present with typical symptoms (53). This difference, we believe, may be attributed in part to sample size and ethnic differences.

Our results highlight the need for healthcare providers to be aware of the sex-specific aspects of managing populations receiving weight-loss therapy during the pandemic and obesity epidemics. At present, there are data available for reference and specific differences from the general population, so it is urgent to increase the attention of this population. Furthermore, the observed sex differences in COVID-19 vulnerability highlight the need to understand better the impact of sex on disease incidence and case fatality rate and to tailor treatment approaches to sex. Ongoing and planned preventive and therapeutic studies should include prospective analyses sensitive to sex.

In our study, we conducted an analysis controlling for center effects, encompassing both primary and secondary outcomes. The results indicated that the impact of center effects on study outcomes was minimal, with relatively limited variability observed across individual centers. Specifically, we found that the size of center effects was not statistically significant, suggesting a minor influence. This finding is, in part, attributable to the fact that the primary study population was drawn from several hospitals in Sichuan, China (Dazhou Central Hospital, Mianyang Central Hospital, and The Third People’s Hospital of Chengdu), totaling 869 individuals. In the same geographic region, similarities in technological advancements and the population undergoing bariatric surgery helped mitigate the potential impact of center effects. However, it is important to note that this conclusion is context-specific to our study. Future research should remain cognizant of the potential variations in center effects in different contexts and continue to address and explore this issue.

This study has some limitations. Firstly, the study was cross-sectional and causality could not be determined. Secondly, due to the inconsistent time intervals between participants’ surgeries and SARS-CoV-2 infection, the study acknowledges the presence of design biases. Additionally, the duration of self-reported severe symptoms by participants might not have been comprehensively considered at the design stage, limiting this open-ended topic to predefined choices. Thirdly, improvement in patients’ comorbidities was not collected, which may somewhat underestimate the benefit of bariatric surgery (54, 55). Fourthly, the investigation depended on self-reported data, thereby potentially susceptible to recall and social desirability bias. Lastly, the risk of finding false positives (Type I error) may increase due to multiple comparisons in our study, necessitating cautious interpretation of our results.

## 5 Conclusion

This cross-sectional study indicates the persistence of sex differences among patients with COVID-19 who have undergone bariatric surgery. Further research is needed to explore the underlying factors contributing to this disparity.

## Data availability statement

The datasets presented in this study can be found in online repositories. The names of the repository/repositories and accession number(s) can be found at: https://figshare.com/s/0ea83dbf1c4cf610037d.

## Ethics statement

The studies involving humans were approved by Ethics Committee of the Third People’s Hospital of Chengdu City. The studies were conducted in accordance with the local legislation and institutional requirements. The participants provided their written informed consent to participate in this study. Written informed consent was obtained from the individual(s) for the publication of any potentially identifiable images or data included in this article. The studies involving humans were approved by the Ethics Committee of the Third People.

## Author contributions

SlW: Conceptualization, Data curation, Investigation, Methodology, Software, Supervision, Writing – original draft, Writing – review & editing. QbJ: Conceptualization, Investigation, Writing – original draft, Writing – review & editing. HW: Investigation. YnY: Investigation. MR: Investigation. JY: Investigation. XyL: Investigation. DL: Conceptualization, Data curation, Investigation, Methodology, Software, Supervision, Writing – original draft, Writing – review & editing.
