# Supplementary material for: Sex differences in patients with COVID-19 after bariatric surgery: a multicenter cross-sectional study
Source: Front Public Health. 2024 Jan 15;11:1293318. doi: 10.3389/fpubh.2023.1293318 (PMC10822963; doi:10.3389/fpubh.2023.1293318)
Supplement: Supplementary file 1 [file Table_1.DOCX]

**Due to privacy concerns, only partial details of the questions are included.**

1. What is your gender?

2. What is your age?

3. What level of education have you attained or received? Please select.

A. Elementary

B. Junior high school

C. High school

D. College

E. Undergraduate

F. Graduate

4. How would you describe your marital status? Please select.

A. Divorce

B. Married

C. Unmarried

5. Do you have a smoking habit? Please select.

A. Current

B. Former

C. Never

6. Do you consume alcohol? Please select.

A. Current

B. Former

C. Never

7. What was your weight before surgery (in kilograms)?

8. What was your BMI before surgery (in kilograms/square meter)?

9. What is your current weight (in kilograms)?

10. What is your current BMI (in kilograms/square meter)?

11. What type of procedure did you undergo? Please select.

A. Sleeve Gastrectomy (SG)

B. Roux-en-Y Gastric Bypass (RYGB)

C. One-anastomosis gastric bypass (OAGB)

D. sleeve gastrectomy with jejunal-jejunal bypass (SG+JJB)

E. Biliopancreatic Diversion with Duodenal Switch (BIDs)

12. How much has your weight changed since the surgery (in kilograms)?

13. How many months elapsed between the time of bariatric surgery and COVID-19 infection?

14. If you forget the specific time, can you provide the accurate month of the surgery date?

15. How many months have passed since your surgery?

16. Do you have hypertension?

17. Do you have hyperlipemia?

18. Do you have coronary heart disease?

19. Do you have gout?

20. Do you have diabetes?

21. Do you have non-alcoholic fatty liver disease?

22. Do you have polycystic ovarian syndrome?

23. Do you have sleep apnea syndrome?

24. Do you have hypothyroidism?

25. Do you have nephrotic syndrome?

26. Do you have tristimania?

27. Do you have osteoarthritis?

28. Do you have acanthosis nigricans?

29. Have you been vaccinated against COVID-19?

30. What is the time of your last dose of vaccination?

31. Have you been infected with COVID-19?

32. How many times have you been infected with COVID-19?

33. Did you experience no symptoms during the infection?

34. What was your temperature during the infection?

35. Did you have a cough during the infection?

36. Did you have a sore throat during the infection?

37. Did you experience diarrhea during the infection?

38. Did you experience vomiting during the infection?

39. Did you feel fatigue during the infection?

40. Did you experience soreness during the infection?

41. Did you experience smell loss during the infection?

42. Did you have nasal congestion and a runny nose during the infection?

43. Did you experience dizziness during the infection?

44. Did you have a headache during the infection?

45. Did you feel chest tightness or pain during the infection?

46. Did you experience dyspnea during the infection?

47. Did you have palpitations of the heart during the infection?

48. Did you feel heart tired during the infection?

49. Did you experience tinnitus in the ears during the infection?

50. Did you have increased appetite during the infection?

51. Did you experience loss of appetite during the infection?

52. Did you experience any other symptoms during the infection?

53. Were the most severe symptoms self-reported during the infection?

54. Was the duration of the worst symptoms self-reported during the infection? Please select.

A. Less than 24 hours

B. One day

C. Two days

D. Three days

E. More than 3 days

55. How many days did it take for you to test negative for COVID-19?

56. Did you take any medication during the infection?

57. Did you engage in exercise during the infection?

58. Did you take multivitamins during the infection?

59. Did you supplement with Vitamin C during the infection?

60. Did you take protein powder during the infection?

61. What is the name of the hospital where you had your bariatric surgery?
