# Supplementary material for: Sex differences in patients with COVID-19 after bariatric surgery: a multicenter cross-sectional study
Source: Front Public Health. 2024 Jan 15;11:1293318. doi: 10.3389/fpubh.2023.1293318 (PMC10822963; doi:10.3389/fpubh.2023.1293318)
Supplement: Supplementary file 2 [file Table_2.DOCX]

**Table S2a. Multivariate logistic regression analysis of clinical characteristics among COVID-19 cases in different sex in China.**

| **No symptoms** | **aPR (95 % CI) ^#^** | ***P* values** |
| --- | --- | --- |
| **Sex** |  |  |
| Males | ref |  |
| Females | 0.40 (0.28 to 0.58) | <0.001 |
| **Age** | 1.02 (0.97 to 1.07) | 0.395 |
| **BMI group** |  |  |
| < 25 | ref |  |
| 25-30 | 2.14 (1.38 to 3.30) | 0.001 |
| 30-35 | 1.38 (0.81 to 2.36) | 0.236 |
| 35-40 | 1.83 (0.39 to 8.68) | 0.447 |
| 40-45 | 9.27 (3.42 to 25.05) | <0.001 |
| 45-50 | 4.87 (1.34 to 17.73) | 0.016 |
| 50-55 | 3.03e-08 (2.89e-09 to 3.17e-07) | <0.001 |
| 55-60 | 2.89e-08 (5.25e-09 to 1.59e-07) | <0.001 |
| **Surgery type** |  |  |
| SG | ref |  |
| RYGB | 1.03e-08 (3.31e-09 to 3.22e-08) | <0.001 |
| Others | 1.10 (0.75 to 1.60) | 0.647 |
| **Educational level** |  |  |
| High school and below | ref |  |
| Junior college | 0.61 (0.32 to 1.18) | 0.138 |
| Undergraduate | 0.57 (0.32 to 0.99) | 0.045 |
| Postgraduate | 0.34 (0.06 to 1.85) | 0.211 |
| **Alcohol consumption** |  |  |
| Current | ref |  |
| Former | 0.72 (0.20 to 2.59) | 0.618 |
| Never | 0.98 (0.46 to 2.06) | 0.949 |
| **Smoking Status** |  |  |
| Current | ref |  |
| Former | 0.63 (0.40 to 1.00) | 0.053 |
| Never | 0.84 (0.36 to 1.94) | 0.685 |
| **Marital Status** |  |  |
| Divorce | ref |  |
| Married | 2.57 (0.58 to 11.42) | 0.215 |
| Unmarried | 2.43 (0.45 to 13.31) | 0.305 |
| **Hypertension (ref: no)** | 0.86 (0.32 to 2.28) | 0.764 |
| **Diabetes (ref: no)** | 1.29 (0.95 to 1.78) | 0.106 |
| **^§^Variance of the group variable** | < 0.001 |  |

**Abbreviations:** PR, Prevalence ratio; CI, confidence interval; BMI,

**#:** adjusted for age (years), BMI group, education level, marital status, smoking status, alcohol consumption, bariatric surgery type, hypertension, and diabetes.

^§^Variance of the group variable indicates the magnitude of variability among individual centers within the groups.

**Table S2b. Multivariate logistic regression analysis of clinical characteristics among COVID-19 cases in different sex in China.**

| **No medication was taken** | **aPR (95 % CI) ^#^** | ***P* values** |
| --- | --- | --- |
| **Sex** |  |  |
| Males | ref |  |
| Females | 0.76 (0.70 to 0.82) | <0.001 |
| **Age** | 1.01 (0.99 to 1.02) | 0.246 |
| **BMI group** |  |  |
| < 25 | ref |  |
| 25-30 | 1.14 (0.91 to 1.42) | 0.266 |
| 30-35 | 0.93 (0.72 to 1.21) | 0.595 |
| 35-40 | 0.86 (0.54 to 1.36) | 0.525 |
| 40-45 | 2.15 (1.25 to 3.68) | 0.005 |
| 45-50 | 0.83 (0.26 to 2.66) | 0.748 |
| 50-55 | 1.29e-07 (3.16e-08 to 5.24e-07) | <0.001 |
| 55-60 | 1.27e-07 (1.25e-08 to 1.29e-06) | <0.001 |
| **Surgery type** |  |  |
| SG | ref |  |
| RYGB | 0.28 (0.09 to 0.83) | 0.023 |
| Others | 1.07 (0.92 to 1.25) | 0.365 |
| **Educational level** |  |  |
| High school and below | ref |  |
| Junior college | 1.16 (0.97 to 1.38) | 0.112 |
| Undergraduate | 1.07 (0.94 to 1.21) | 0.292 |
| Postgraduate | 1.13 (0.85 to 1.49) | 0.401 |
| **Alcohol consumption** |  |  |
| Current | ref |  |
| Former | 1.06 (0.68 to 1.64) | 0.794 |
| Never | 0.84 (0.50 to 1.40) | 0.503 |
| **Smoking Status** |  |  |
| Current | ref |  |
| Former | 1.06 (0.77 to 1.45) | 0.735 |
| Never | 0.96 (0.60 to 1.54) | 0.883 |
| **Marital Status** |  |  |
| Divorce | ref |  |
| Married | 1.90 (1.30 to 2.78) | 0.001 |
| Unmarried | 2.16 (1.43 to 3.28) | <0.001 |
| **Hypertension (ref: no)** | 1.06 (0.76 to 1.46) | 0.741 |
| **Diabetes (ref: no)** | 1.03 (0.59 to 1.79) | 0.925 |
| **^§^Variance of the group variable** | < 0.001 |  |

**Abbreviations:** PR, Prevalence ratio; CI, confidence interval; BMI,

**#:** adjusted for age (years), BMI group, education level, marital status, smoking status, alcohol consumption, bariatric surgery type, hypertension, and diabetes.

^§^Variance of the group variable indicates the magnitude of variability among individual centers within the groups.

**Table S2c. Multivariate logistic regression analysis of clinical characteristics among COVID-19 cases in different sex in China.**

| **Number of infections with COVID-19** | **PR (95 % CI) ^#^** | ***P* values** |
| --- | --- | --- |
| **Sex** |  |  |
| Males | ref |  |
| Females | 0.39 (0.20 to 0.74) | 0.004 |
| **Age** | 0.94 (0.92 to 0.96) | < 0.001 |
| **BMI group** |  |  |
| < 25 | ref |  |
| 25-30 | 0.95 (0.53 to 1.69) | 0.853 |
| 30-35 | 0.53 (0.29 to 0.96) | 0.037 |
| 35-40 | 0.78 (0.25 to 2.38) | 0.659 |
| 40-45 | NA | NA |
| 45-50 | NA | NA |
| 50-55 | NA | NA |
| 55-60 | NA | NA |
| **Surgery type** |  |  |
| SG | ref |  |
| RYGB | 1.33 (0.71 to 2.49) | 0.380 |
| Others | 1.14 (0.48 to 2.74) | 0.764 |
| **Educational level** |  |  |
| High school and below | ref |  |
| Junior college | 1.03 (0.47 to 2.25) | 0.947 |
| Undergraduate | 0.72 (0.19 to 2.76) | 0.627 |
| Postgraduate | 1.01 (0.33 to 3.09) | 0.986 |
| **Alcohol consumption** |  |  |
| Current | ref |  |
| Former | 6.90e+08 (3.66e+07 to 1.30e+10) | < 0.001 |
| Never | 3.90e+08 (2.19e+07 to 6.96e+09) | < 0.001 |
| **Smoking Status** |  |  |
| Current | ref |  |
| Former | 0.16 (0.01 to 4.24) | 0.277 |
| Never | 0.49 (0.15 to 1.58) | 0.235 |
| **Marital Status** |  |  |
| Divorce | ref |  |
| Married | 1.16 (0.08 to 16.09) | 0.913 |
| Unmarried | 1.09 (0.05 to 22.90) | 0.957 |
| **Hypertension (ref: no)** | 4.00 (1.75 to 9.14) | 0.001 |
| **Diabetes (ref: no)** | 2.30 (1.18 to 4.49) | 0.014 |
| **^§^Variance of the group variable** | < 0.001 |  |

**Abbreviations:** PR, Prevalence ratio; CI, confidence interval; BMI,

**#:** adjusted for age (years), BMI group, education level, marital status, smoking status, alcohol consumption, bariatric surgery type, hypertension, and diabetes.

^§^Variance of the group variable indicates the magnitude of variability among individual centers within the groups.
