# Supplementary material for: Sex differences in patients with COVID-19 after bariatric surgery: a multicenter cross-sectional study
Source: Front Public Health. 2024 Jan 15;11:1293318. doi: 10.3389/fpubh.2023.1293318 (PMC10822963; doi:10.3389/fpubh.2023.1293318)
Supplement: Supplementary file 3 [file Table_3.DOCX]

**Table 3. Sex differences in self-reported symptoms of COVID-19.**

| \|  \| **Male** \| **Female** \| **Total** \|  \| \| --- \| --- \| --- \| --- \| --- \| \| **Symptoms** \| **(N = 282)** \| **(N = 852)** \| **(N = 1134)** \| ****p*-value** \| |
| --- | --- | --- | --- | --- | --- | --- | --- | --- | --- | --- |
| \| **Without any symptoms** \| 21 (7.4%) \| 27 (3.2%) \| 48 (4.2%) \| 0.002 \| \| --- \| --- \| --- \| --- \| --- \| \| **Cough** \| 142 (50.4%) \| 573 (67.3%) \| 715 (63.1%) \| <0.001 \| \| **Sore throat** \| 92 (32.6%) \| 417 (48.9%) \| 509 (44.9%) \| <0.001 \| \| **Diarrhea** \| 30 (10.6%) \| 113 (13.3%) \| 143 (12.6%) \| 0.250 \| \| **Vomiting** \| 14 (5.0%) \| 74 (8.7%) \| 88 (7.8%) \| 0.043 \| \| **Fatigue** \| 133 (47.2%) \| 445 (52.2%) \| 578 (51.0%) \| 0.140 \| \| **soreness** \| 145 (51.4%) \| 561 (65.8%) \| 706 (62.3%) \| <0.001 \| \| **smell loss** \| 80 (28.4%) \| 340 (39.9%) \| 420 (37.0%) \| 0.001 \| \| **Nasal congestion and runny nose** \| 102 (36.2%) \| 409 (48.0%) \| 511 (45.1%) \| 0.001 \| \| **Dizziness** \| 72 (25.5%) \| 288 (33.8%) \| 360 (31.7%) \| 0.010 \| \| **Headache** \| 94 (33.3%) \| 350 (41.1%) \| 444 (39.2%) \| 0.021 \| \| **Chest tightness or pain** \| 29 (10.3%) \| 94 (11.0%) \| 123 (10.8%) \| 0.726 \| \| **Dyspnea** \| 13 (4.6%) \| 65 (7.6%) \| 78 (6.9%) \| 0.082 \| \| **Palpitations of heart** \| 14 (5.0%) \| 69 (8.1%) \| 83 (7.3%) \| 0.080 \| \| **Heart tired** \| 18 (6.4%) \| 92 (10.8%) \| 110 (9.7%) \| 0.030 \| \| **Tinnitus in the ears** \| 21 (7.4%) \| 68 (8.0%) \| 89 (7.8%) \| 0.772 \| \| **Increased appetite** \| 20 (7.1%) \| 104 (12.2%) \| 124 (10.9%) \| 0.017 \| \| **Loss of appetite** \| 51 (18.1%) \| 286 (33.6%) \| 337 (29.7%) \| <0.001 \| |

**Abbreviations:**

^*^*P* value by chi-square test for categorical variables, and Fisher exact probability method test for categorical variables when the theoretical frequency in the count data is less than or equal to 5.
