# Supplementary material for: Sex differences in patients with COVID-19 after bariatric surgery: a multicenter cross-sectional study
Source: Front Public Health. 2024 Jan 15;11:1293318. doi: 10.3389/fpubh.2023.1293318 (PMC10822963; doi:10.3389/fpubh.2023.1293318)
Supplement: Supplementary file 5 [file Data_Sheet_1.PDF]

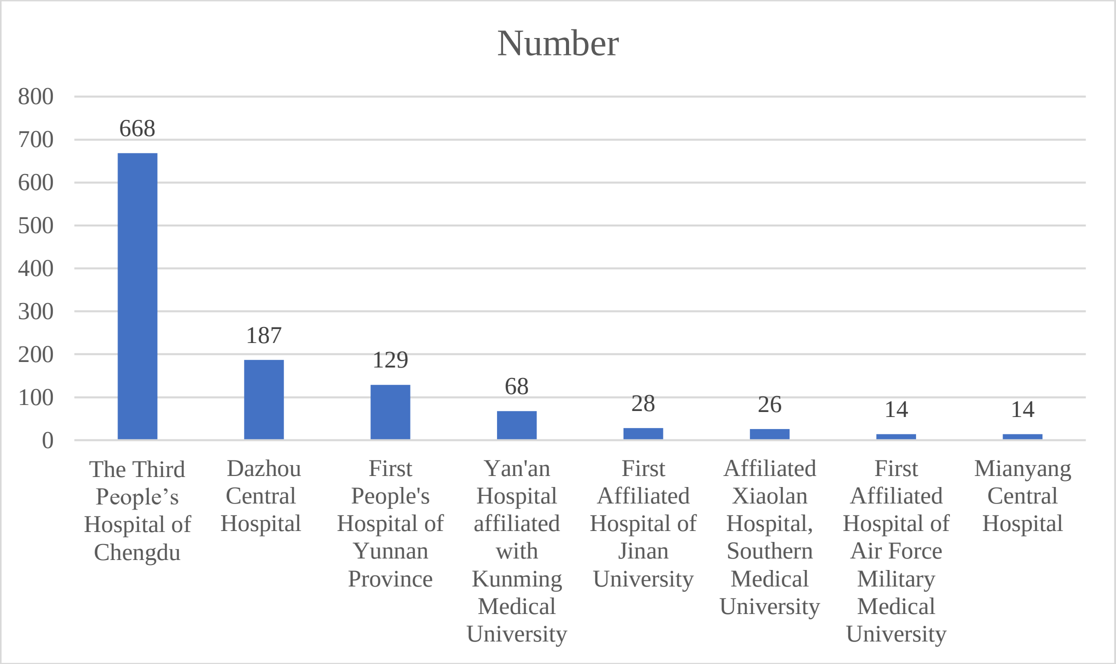

Figure S1. A bar chart illustrates the number of participants from each center included in the analysis.
